# Communication Skills in Group Psychoeducation

**DOI:** 10.1192/bjo.2022.274

**Published:** 2022-06-20

**Authors:** Muhammad Ayub, M. Iqbal Afridi

**Affiliations:** Dept. of Psychiatry & Behavioural Sciences, JPMC (Jinnah Postgraduate Medical Centre), Karachi-75510, Pakistan

## Abstract

**Aims:**

To Improve the mental health of psychiatric inpatients and caregivers. To improve communication skills of postgraduate trainees.

**Methods:**

Setting; Consented, monitoring and observation of communication skills during weekly, inpatient Psychoeducation sessions at Department of Psychiatry and Behavioural Sciences, JPMC, Karachi.

Data collection; Retrospective, communication skill records of postgraduate trainees from last 10 sessions from July 2019 to October, 2020. Based on a 13-items self-made questionnaire for communication skill. The overall communication skills of each postgraduate trainee were recorded from excellent, very good, good, improvement needed and lots of improvement needed category based on their performance.

**Results:**

Current practice showed that communication skills of 70% of postgraduate trainees were recorded as very good communication skill, 30% into excellent while none was noticed in another category.

**Re-audit:**

: It was started soon after implementation of action plan from November, 2019 to January, 2020, with monitoring of weekly inpatient psychoeducation sessions similarly as done previously. The result of reaudit concluded significant improvement in individual and overall communication skill which were recorded as very good 50% and excellent 50% and none had other poorer categories of communications Skills.

**Conclusion:**

Individual feedbacks to doctors immediately after the psychotherapy session according to the audit tool questionnaire to improve current communication skills.

